# Lacertus syndrome: a ten year analysis of two hundred and seventy five minimally invasive surgical decompressions of median nerve entrapment at the elbow

**DOI:** 10.1007/s00264-023-05709-w

**Published:** 2023-02-09

**Authors:** Elisabet Hagert, Ulrika Jedeskog, Carl-Göran Hagert, Theodorakys Marín Fermín

**Affiliations:** 1 Aspetar Orthopaedic and Sports Medicine Hospital, Sports City Street, Inside Aspire Zone, Al Buwairda St, 29222 Doha, Qatar; 2 Dept of Clinical Science and Education, Karolinska Institutet, Sodersjukhuset, Stockholm, Sweden; 3 Arcademy, Sophiahemmet Hospital, Stockholm, Sweden; 4 Bjärred, Sweden

**Keywords:** Carpal tunnel syndrome, Mononeuropathies, Median neuropathy, Nerve compression syndromes, Peripheral nerves

## Abstract

**Purpose:**

This study aims to assess the clinical presentation and surgical outcomes of lacertus syndrome (LS) and concomitant median nerve entrapments.

**Methods:**

A retrospective study of prospectively collected data was conducted on patients undergoing lacertus release (LR) from June 2012 to June 2021. Available DASH (Disability of the Arm Shoulder Hand questionnaire) scores and post-operative Visual Analogue Scale (VAS) of pain, numbness, subjective satisfaction with surgical outcome, and intra-operative return of strength were analyzed.

**Results:**

Two-hundred-seventy-five surgical cases were identified of which 205 cases (74.5%) underwent isolated LR, and 69 cases (25.1%) concomitant lacertus and carpal tunnel release. The three most common presenting symptoms in LS patients were loss of hand strength (95.6%), loss of hand endurance/fatigue (73.3%), and forearm pain (35.4%). Numbness in the median nerve territory of the hand was found in all patients with combined LS and carpal tunnel syndrome. Quick-DASH significantly improved (pre-operative 34.4 (range 2.3–84.1) to post-operative 12.4 (range 0–62.5), *p* < 0.0001) as did work and activity DASH (*p* < 0.0001). The postoperative VAS scores were pain VAS 1.9 and numbness VAS 1.8. Eighty-eight percent of patients reported good/excellent satisfaction with the surgical outcome. Intra-operative return of strength was verified in 99.2% of cases.

**Conclusion:**

LS is a common median nerve compression syndrome typically presenting with loss of hand strength and hand endurance/fatigue. Minimally invasive LR immediately restores hand strength, significantly improves DASH scores, and yields positive outcomes regarding VAS pain, numbness, and subjective satisfaction with surgery in patients with proximal median nerve entrapment at a minimum six month follow-up.

**Supplementary Information:**

The online version contains supplementary material available at 10.1007/s00264-023-05709-w.

## Introduction

Proximal median nerve entrapment (PMNE) has attracted the interest of hand surgeons over the last decade [1]. Henrik Seyffarth first described it as pronator syndrome in 1951 [2], a nerve compression between the humeral and ulnar head of the pronator teres muscle, usually presenting a fibrous band between them. However, recent research has challenged which structure is primarily responsible for compression and the extent of the surgical release required to relieve symptoms [3, 4].

**Fig. 1:**
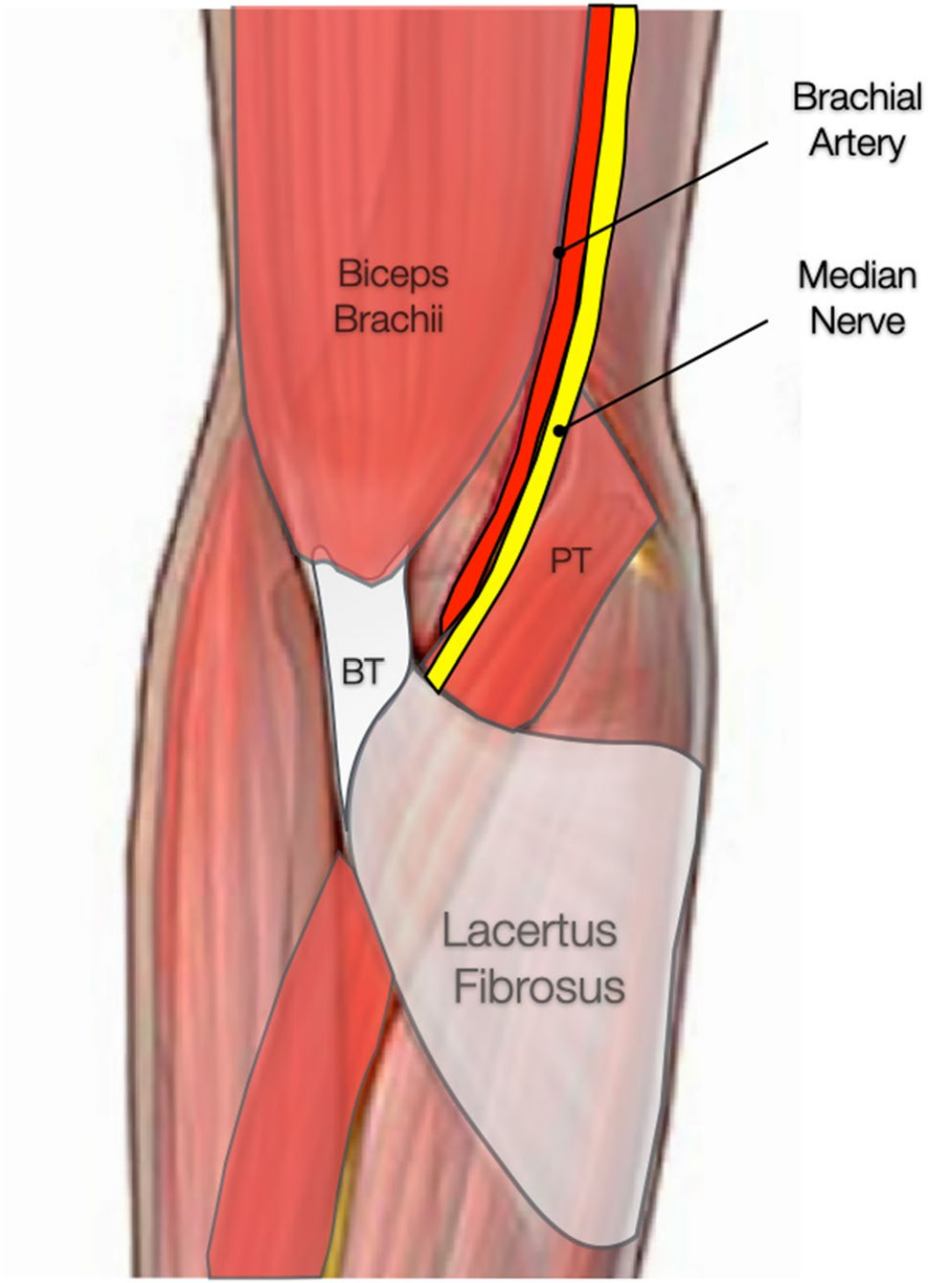
Anatomy of the lacertus fibrosus, also known as the bicipital aponeurosis, expanding from the common distal tendon of the biceps brachii (BT) to cross over the proximal flexor-pronator teres (PT) mass. The brachial artery and median nerve pass under the lacertus fibrosus at the level of the cubital fossa

The lacertus fibrosus (LF) is an aponeurosis originating from the medial border of the distal biceps brachialis tendon, directed medially and distally, and in direct contact with the median nerve in almost half of individuals, crossing over the common flexor muscle mass and blending with its fascia [5, 6] (Fig. 1). Its dynamic biomechanical role in force transmission during elbow flexion, lever arm adjustment, and supination restraint could also explain its dynamic compression over the median nerve [4, 5]. In fact, due to the nerve topography and dynamic nature of entrapment caused by the LF, commonly known as *lacertus syndrome* (LS), its symptomatology often goes unnoticed [7, 8].

Additionally, the literature on concomitant carpal tunnel syndrome (CTS) and PMNE remain scarce, reporting a prevalence ranging from 6 to 13% [9–11]. The underdiagnosis of the LS and the unsatisfactory outcomes after carpal tunnel release in double-crush syndromes demand an in-depth analysis of this entity’s current diagnostic and treatment approach [1, 11]. The aim of this study was to assess the clinical presentation and surgical outcomes of LS and concomitant median nerve entrapments.

## Materials and methods

This study involving human participants was in accordance with the ethical standards of the 1964 Helsinki Declaration and its later amendments. Ethics committee approval and due consent were also obtained. A retrospective study of prospectively collected data was conducted on the patient registry from June 2012 to June 2021. Patients were included for final evaluation if they had undergone surgical decompression of the median nerve at the level of the LF (lacertus release), with or without simultaneous concomitant carpal tunnel or other median nerve releases. Patients surgically treated with concomitant peripheral nerve compressions other than the median nerve were excluded.

Medical charts were reviewed, and data on sex, age, occupation, hand dominance, reported subjective symptoms, surgical treatment, and intra-operative return of strength were collected. Available pre-and post-operative quick-DASH (Disability of the Arm Shoulder Hand questionnaire) with work and activity sub-scores and post-operative Visual Analogue Scale (VAS) of pain, numbness, subjective satisfaction with the surgical outcome, and intra-operative return of strength were analyzed.

### Lacertus syndrome diagnosis

The diagnosis of LS was based on thorough patient history and clinical examination, including the previously described clinical triad of combining upper extremity manual muscle testing revealing weakness in the flexor carpi radialis (FCR), flexor pollicis longus (FPL), and flexor digitorum profundus to the index finger (FDP II), provocative sensory testing (scratch collapse test, SCT), and the presence of pain and/or positive Tinel’s test at the level of nerve compression (Online Resource 1) [4]. For the LS, pain is present at the level of the median nerve under the LF. Concomitant CTS was defined by a positive Tinel’s test and other provocative maneuvers. No electromyographic studies were implemented in diagnosing LS.

### Surgical technique

**Fig. 2:**
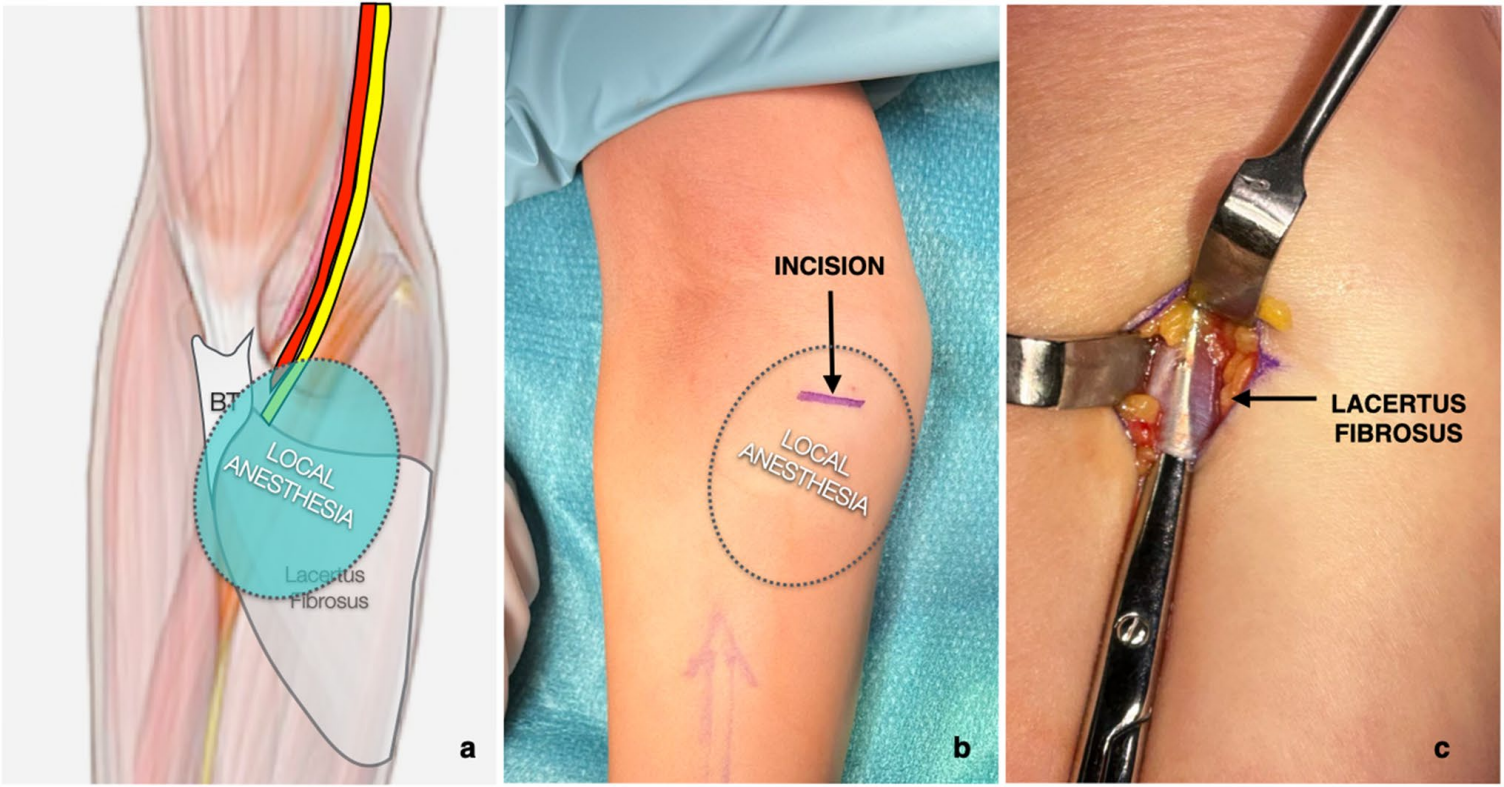
Illustration of the minimally invasive surgical technique for lacertus release. **a** 20–30 cc of 1% lidocaine-epinephrine solution is injected subcutaneously over the course of the lacertus fibrosus; **b** a 1.5–2-cm transverse skin incision is placed over the lacertus fibrosus, in the ulnovolar, and proximal aspect of the forearm; **c** following blunt dissection to forearm fascia, the lacertus fibrosus is easily identified and divided

Decompression of the median nerve at the level of the LF (lacertus release) followed a technique previously described [4] (Fig. 2). It was performed under wide-awake, local anaesthesia, and no tourniquet (WALANT) whenever feasible [12, 13]. The advantage of doing this procedure in an awake and cooperative patient is that intra-operative return of muscle power can be monitored, and possible further release if required, can be done immediately.

### Outcome measures

Outcome measures were prospectively collected via post six months post-operatively by an investigator outside the surgical team. A second letter was sent nine months post-operatively if no answer was retrieved after the first. The primary outcomes were the pre- and post-operative quick-DASH with work and activity sub-scores. The DASH questionnaire is a validated tool for standard assessment of the impact on the function of various musculoskeletal diseases and injuries in the upper extremity; scores range from 0 to 100, with higher ranking indicating worse symptoms [14, 15]. Secondary outcomes included the post-operative VAS for pain, numbness, subjective satisfaction with the surgery (VAS 0-10) [4], and intra-operative return of strength.

### Statistical analyses

Pre-operative and post-operative scores were compared using paired two-tailed Student’s *t*-test. *P* values of < 0.05 were considered statistically significant. SPSS V.19 was used to perform statistical analysis. Results were presented as mean, ranges, median, and interquartile ranges.

## Results

A total of 388 lacertus releases were identified in the ten year period. The final cohort included 275 lacertus releases in 245 patients after excluding patients who underwent simultaneous nerve decompressions of other nerves (radial nerve, *N* = 77; ulnar nerve, *N* = 39 patients). Of these, 205 cases (74.5%) were isolated lacertus release, 69 cases (25.1%) were concomitant lacertus and carpal tunnel release, and one had concomitant lacertus and median nerve release at the superficialis arcade. Twenty-six patients (12.7%) who underwent isolated lacertus release had prior carpal tunnel release with incomplete resolution of symptoms. Patients’ demographics are summarized in Table 1.

**Table 1:** Patient demographics

Patient variables	No.	
Age (year), mean (range)	47 (19–73)	
Sex, no. (%)	Female = 137 (49.8%)	Male = 138 (50.2%)
Arm affected, no. (%)	Dominant = 184 (66.9%)	Non-dominant = 91 (33.1%)
Manual worker, no. (%)	71 (25.8%)	
Pronated work, no. (%)	222 (80.7%)	

### Presenting symptoms

**Fig. 3:**
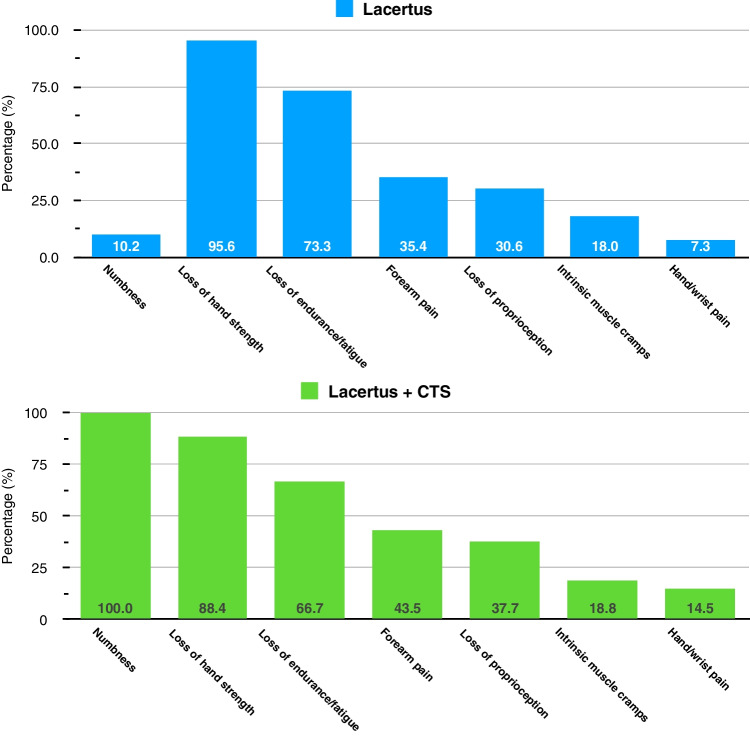
Presenting symptoms of lacertus syndrome (top) and combined lacertus and carpal tunnel syndromes (bottom). The three most common presenting symptoms in lacertus syndrome patients were loss of hand strength (95.6%), loss of hand endurance/fatigue (73.3%), and forearm pain (35.4%). Numbness in the median nerve territory of the hand was found in all of the patients with combined lacertus and carpal tunnel syndromes

The three most common presenting symptoms in LS patients were loss of hand strength (95.6%), loss of hand endurance/fatigue (73.3%), and forearm pain (35.4%) (Fig. 3). Numbness in the median nerve territory of the hand was found in all patients with combined LS and CTS. In contrast, only 10% of the patients with LS without previous history of carpal tunnel release presented with numbness, mainly within the palmar branch of the median cutaneous nerve or at the tips of the first to third digits.

### Surgical outcomes

**Fig. 4:**
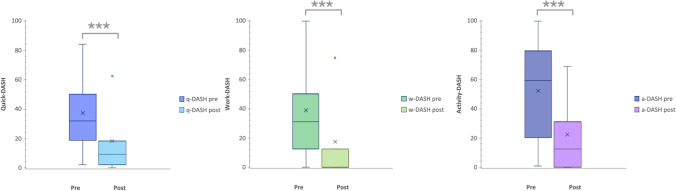
Box-and-whisker plots showing the results of pre-operative and post-operative quick-DASH, work-DASH, and activity-DASH scores. The boxes show the median and interquartile range, and the whiskers show the 25th and 75th percentiles. Cross marks the average, and dot indicates outliers outside the whiskers. ***Statistically significant changes in pre- and post-operative scores (*p* < 0.0001)

Pre-operative quick-DASH with work and activity sub-scores were obtained from 227 patients. Of these, 136 patients (60%) completed the post-operative follow-up questionnaires. The average pre-operative quick DASH was 34.4 (range 2.3–84.1), work DASH 34.2 (0–100), and activity DASH 53.8 (range 0–100). The average post-operative quick DASH was 12.4 (range 0–62.5, mean difference 22), which is a statistically significant reduction (*p* < 0.0001). Similarly, the work and activity DASH subscores were significantly reduced (*p* < 0.0001) to 8.4 (0–75) and 19.5 (0–68.7), respectively (Fig. 4).

The mean post-operative VAS scores were as follows: pain VAS 1.9; numbness VAS 1.8; satisfaction with the surgical outcome VAS 8.5. Eighty-eight percent of patients reported good/excellent satisfaction with the surgical outcome. Only four patients were unsatisfied with the surgical outcome (1.6%), all of whom were later found to have a residual median nerve compression at the superficialis arcade that required revision surgery.

Two hundred fifty cases (91%) were done using WALANT anaesthesia which allows immediate evaluation of return of power in the FCR, FPL, and FDP II following lacertus release (Online Resource 2). Intraoperative return of strength was verified in 99.2% (248) of the patients. The two where power was not back on the table were with a complete palsy of the anterior interosseous nerve (FPL and FDP II palsy), and power was restored within eight weeks after surgery.

## Discussion

The main findings of this study are that median nerve compression at the LF is a common entrapment syndrome and that lacertus release significantly improves quick-DASH score and subscores, VAS pain, numbness, and subjective satisfaction with the surgery at a minimum six month follow-up. Furthermore, it immediately restores hand strength in 99.2% of the patients.

Systematic clinical examination of strength in peripheral nerve entrapments has yielded a higher prevalence of dynamic compressions with no electromyographic changes (Sunderland zero compression neuropathies) and should thus be performed routinely [16–21]. The results of the present study show that patients with isolated LS typically present with complaints of loss of hand strength and loss of hand endurance/fatigue. The presence of associated median territory numbness ought to raise suspicion about concomitant CTS.

Correspondingly, patients with incomplete symptomatic relief after carpal tunnel release should be assessed to rule out concomitant LS, as they represented a not negligible 12.7% of the cases in this series. Other authors, like Binder et al. [16], found in a retrospective cohort study comprising 26 patients with pronator syndrome that 38.5% had a previous history of carpal tunnel release. Similarly, El-Haj et al. [18], in their retrospective study, including 27 patients with PMNE release, reported the same scenario in 48% of them. The wide range prevalence of unsatisfactory symptom relief after carpal tunnel release among these series can be explained by the lack of standardized diagnostic pathways, as the resulting PMNE motor deficit continues to be underdiagnosed and differential diagnoses still focus on the sensory deficits missing primary double-crush syndromes [10, 22].

Previous studies on PMNE have yielded satisfactory surgical outcomes. Binder et al. reported significant DASH (preop 48.7 ± 15.5 to postop 22.8 ± 18.4, *p* < 0.01) and VAS score (preop 8 ± 1 to postop 0.7 ± 1.4, *p* < 0.01) improvement after open PMNE release in twelve patients at a mean 75 months follow-up. Lee et al. [23] had similar outcomes implementing endoscopically assisted pronator syndrome decompression in 12 patients with an average of 51 points DASH score improvement (preop 57 to postop 6, *p* < 0.05) at a mean 22 months follow-up. Similarly, Apard et al. [17] presented a prospective cohort study of 15 patients undergoing ultrasound-guided percutaneous lacertus release, showing significant VAS score improvement (preop 6.2 to postop 0.6, *p* < 0.001) at four weeks post-operatively and immediate post-operative muscle power restoration in all patients.

The present study shows comparable outcomes by implementing an isolated mini-open lacertus release and exceeding the minimum clinically important difference in the quick-DASH score [24, 25]. However, it is still debatable which is the primary structure responsible for PMNE, with the lack of studies comparing surgical approaches partaking in it. This study contributes to this debate by revealing an immediate post-operative return of strength in 99.2% of the patients through the isolated release of the LF, thus highlighting this structure’s role in median nerve entrapment syndromes.

In fact, in the present cohort of 275 cases, only two patients were intra-operatively found to have a course of the median nerve that went between the ulnar and humeral heads of the pronator teres and, thus, could be defined as pronator syndromes in accordance with the original definition by Seyffarth [2]. Clinically, these two patients did not differ from the patients where the LF was the primary compressing structure. Additionally, one patient had concomitant compression at the superficialis arcade, and four revision procedures were done to release the median nerve at this level. For the superficialis arcade compression, the symptoms were that of pain approximately 3 cm distal of the LF as well as weakness in the flexor digitorum superficialis to the middle and ring fingers, a weakness not seen if the compression is at the LF alone. Although pronator involvement was exceedingly rare in our cohort, it is interesting to note that 222 patients (80.7%) reported having work that consisted of pronation for a minimum of five hours per day. It may thus be suggested that the vast majority of PMNE are not pronator syndrome but rather syndrome of pronation.

Pre-operative evaluation of the median nerve with ultrasound may be considered to distinguish between these rare but potentially differential diagnoses of PMNE sites. Ultrasound is a reliable imaging tool for diagnosing carpal tunnel syndrome [26] and is also proposed as a treatment modality for ultrasound-guided median nerve release both at the carpal tunnel and LF [17, 27, 28]. To date, however, there are no standardized protocols for median nerve ultrasound diagnostics proximal to the carpal tunnel. Future studies should aim to investigate normal and pathological median nerve ultrasound appearance from the distal upper arm (Struther’s arcade) to the mid-forearm (superficialis arcade) with regard to nerve size and structural integrity [29].

The present study certainly has some limitations. First, the short-term follow-up of patients, which is due to a fast recovery and symptomatic improvement after the surgical procedure, stops patients from complying with future appointments. Second is the lack of electromyographic assessment. However, as reported by other authors, electromyography has a limited role in diagnosing this clinical entity due to the dynamic nature of the median nerve entrapment [19–21, 30]. Lastly, the qualitative examination of the loss of strength was compensated with a comparative intraoperative strength assessment pre- and post-release. Future studies should focus on the prevalence of this syndrome and dynamic quantitative tests for its diagnostic workup.

## Conclusion

Patients with isolated LS typically present with loss of hand strength and hand endurance/fatigue. Minimally invasive lacertus release immediately restores hand strength, significantly improves quick-DASH score and subscores, and yields positive outcomes regarding VAS pain, numbness, and subjective satisfaction with the surgery in patients with PMNE at a minimum six month follow-up.

## Supplementary information

Supplementary file 1 Online Resource 1: Video of the clinical examination of the lacertus syndrome.

Supplementary file 2 Online Resource 2: Video of the preoperative and intraoperative measurement of strength before and after lacertus release.

## Data Availability

The data underlying this article are available in the article and its online supplementary material.

## Abbreviations

CTS: carpal tunnel syndrome
DASH: Disability of the Arm Shoulder Hand Questionnaire
FCR: flexor carpi radialis
FDP II: flexor digitorum profundus to the index finger
FPL: flexor pollicis longus
LF: lacertus fibrosus
LS: lacertus syndrome
PMNE: proximal median nerve entrapment
VAS: visual analog scale
WALANT: wide-awake, local anesthesia, no tourniquet
